# HIV-1 Ribonuclease H: Structure, Catalytic Mechanism and Inhibitors

**DOI:** 10.3390/v2040900

**Published:** 2010-03-30

**Authors:** Greg L. Beilhartz, Matthias Götte

**Affiliations:** Department of Microbiology and Immunology, McGill University, Lyman Duff Medical Building (D6), 3775 University St., Montreal, QC, H3A 2B4, Canada

**Keywords:** HIV, reverse transcriptase, RNase H, inhibitors, drug resistance

## Abstract

Since the human immunodeficiency virus (HIV) was discovered as the etiological agent of acquired immunodeficiency syndrome (AIDS), it has encouraged much research into antiviral compounds. The reverse transcriptase (RT) of HIV has been a main target for antiviral drugs. However, all drugs developed so far inhibit the polymerase function of the enzyme, while none of the approved antiviral agents inhibit specifically the necessary ribonuclease H (RNase H) function of RT. This review provides a background on structure-function relationships of HIV-1 RNase H, as well as an outline of current attempts to develop novel, potent chemotherapeutics against a difficult drug target.

## 1. Introduction

**Figure 1.**
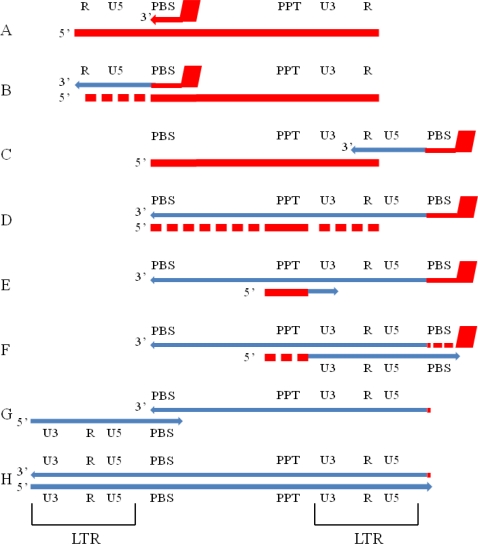
Schematic of the process of reverse transcription. **A** The viral RNA genome is shown as a thick red line. Reverse transcription is initiated by the binding of an endogenous tRNA^lys3^ molecule to the primer binding site (PBS) on the genome. **B** RT elongates the tRNA primer to the 5′ end of the genome, creating a fragment called (−)-strand strong stop DNA ((−)ssDNA). The RNase H activity of RT concomitantly degrades the RNA genome during DNA synthesis. The degradation of the 5′ end of the genome is necessary for (−)-strand transfer, and failure to degrade the RNA at this point results in the arrest of reverse transcription. **C** First or (−)-strand transfer. The (−)ssDNA fragment dissociates from the PBS sequence and re-associates with the repeat (R) sequence at the 3′ end of the genome. This step is capable of both intrastrand and interstrand transfer. **D** Continuation of (−)-strand DNA synthesis. RT extends the 3′ end of the (−)ssDNA fragment toward the PBS sequence, while the RNase H activity concomitantly degrades the RNA genome, which the exception of the polypurine tract (PPT). **E** The PPT is used as the primer for the initiation of (+)-strand DNA synthesis. The PPT primer is extended by the RT polymerase activity. **F** After approximately 12 nucleotides have been added, the PPT primer is removed by RNase H activity. The nascent (+)-strand DNA is extended to the 5′ end of the (−)-strand DNA, copying the PBS sequence from the tRNA that is still associated with the (−)-strand DNA. Here, the tRNA is removed by the RNase H activity, leaving a single ribonucleotide (rA) at the 3′ end of the U5 sequence (shown in red). **G** In the second, or (+)-strand transfer, the PBS sequences on both strands associate. This step occurs predominantly in an intrastrand fashion. **H** Both DNA strands are extended to the ends of their templates, forming the provirus that is ready to be integrated into the host genome by integrase. The long terminal repeats (LTRs) that are formed as a result of reverse transcription are shown.

The human immunodeficiency virus type 1 (HIV-1) replicates through the process of reverse transcription. This is accomplished through a virally-encoded, heterodimeric reverse transcriptase (RT) enzyme comprised of a catalytic p66 subunit, and a smaller p51 subunit which plays a mainly structural role [1]. The p66 subunit is subdivided into three domains: the N-terminal polymerase domain, the C-terminal ribonuclease (RNase) H domain, and connection domain that links the two functional regions. The process of reverse transcription involves the copying of the single-stranded RNA of the viral genome into double-stranded DNA (Figure 1).

This requires that RT is able to act at times as a RNA-dependent DNA polymerase, a DNA-dependent DNA polymerase, and as an RNase H that cleaves the RNA of RNA/DNA hybrids. These activities are coordinated temporally and spatially. As all three of these processes are absolutely required for the successful completion of reverse transcription, and, as such, virus replication, RT has been amongst the most successful drug targets at this point. However, there exists a bias towards RT inhibitors that interfere with the polymerase functions of RT [2]. Despite the lack of RNase H inhibitors that have advanced into the clinic, much progress has been made towards a better understanding of the HIV-1 RT-associated RNase H structure and function, as well as homologous enzymes. This review discusses the structure and function of HIV-1 RNase H with emphasis on the cross-talk between the polymerase and RNase H active sites, RNase H inhibitors, and the role of RNase H in drug resistance to established antivirals.

## 2. RNase H Structure and Function

**Figure 2.**
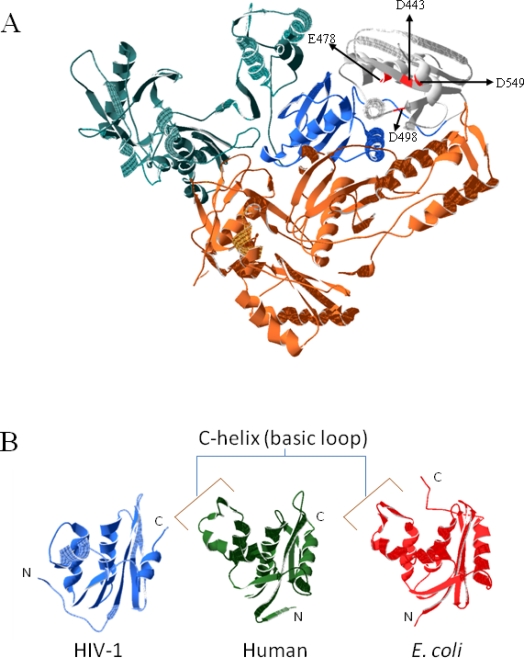
**A** Crystal structure of HIV-1 RT (PDB code: 1RTD. The p51 subunit is shown in orange, while the p66 subunit is divided into the fingers (cyan), connection (blue) and RNase H (grey) subdomains. The residues of the conserved DEDD motif are shown and red and marked with arrows. **B** Crystal structures of the RNase H domain of HIV-1 RT (PDB code:1RTD) [5], human RNase H1 (PDB code: 2QKB) [12] and *E. coli* RNase H1 (PBD code: 1WSJ). All three show the same mixed beta-sheet with asymmetric alpha helices, while the human and *E. coli* RNases H contain the C-helix, or basic loop.

RNase H functions as an endonuclease that specifically cleaves the RNA moiety of RNA/DNA hybrids. In the case of HIV-1, RNase H non-specifically degrades the (+)-strand RNA genome, while specifically removing the (−)-strand tRNA primer and creating and removing the (+)-strand or PPT primer during reverse transcription (Figure 1). RNases H exist both as a free enzyme (*E. coli* RNase H1) and as a domain in a larger enzyme as in HIV-1 RT. The structure of both remains highly conserved, albeit with some crucial differences. In the case of HIV-1 RT, the RNase H domain constitutes the C-terminus of the p66 subunit, which is missing in p51, due to cleavage by the viral protease [3–7]. The folded structure of the HIV-1 RNase H domain takes the form of a 5-stranded mixed beta-sheet flanked by four alpha helices in an asymmetric distribution (Figure 2) [3]. The structure is homologous to other retroviral RNases H such as murine leukemia virus (MLV) [8] and avian sarcoma leukemia virus (ASLV), and both prokaryotic (*E. coli, B. halodurans*) [9–11] and eukaryotic (human) [12] RNases H, and is part of the superfamily of polynucleotidyl transferases. A notable difference between the various RNase H proteins is the presence or absence of the C-helix (present in *E. coli*, MLV and human RNases H, absent in HIV-1, *B. halodurans* and ASLV RNases H), a positively charged alpha helix also referred to as the basic loop, protrusion, or handle, and is thought to aid in substrate binding (Figure 2). Interestingly, deletion of the basic loop in *E. coli* inhibits but does not abolish activity [13], although the isolated RNase H domain of MLV with the basic loop deleted is not active [8]. However, if the basic loop in *E. coli* RNase H is inserted into an isolated inactive HIV-1 RNase H domain, Mn^2+^-dependent activity is partially restored [14]. In HIV-1 RT, the connection subdomain contains a small loop (residues K353 to T365) that contains several basic residues and is structurally located at the exact position of the *E. coli* and MLV basic loops, and is thought to compensate for the lack of the C-helix [8,12] since RNase H activity was restored to an inactive isolated domain with the addition of the p66 connection domain *in trans* [15].

**Figure 3.**
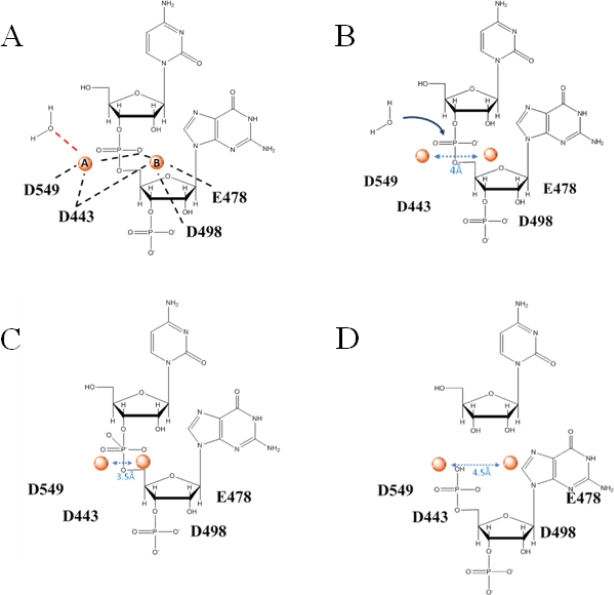
The chemistry of RNase H cleavage is believed to be a two-metal ion mechanism. **A** Two divalent metal ions (red spheres, marked A and B) are coordinated by the active site residues D549, D443, D498 and E478 approximately 4Å apart. Metal ion A activates a water molecule. **B** The activated water molecule carries out a nucleophilic attack (blue arrow) driving the phosphoryl transfer reaction. **C** In the putative transition state, the metal ions move toward each other to bring the nucleophile within range of the scissile phosphate. **D** The reaction products consist of a 3′ OH group and a 5′ phosphate group, and the metal ions are again likely to be re-positioned.

The active center of the HIV-1 RNase H domain contains a highly conserved DEDD motif (D443, E478, D498, D549), which coordinates two divalent cations required to hydrolyze the RNA substrate. Magnesium is likely the physiologically relevant ion; however, HIV-1 RNase H will tolerate manganese, cobalt, and other cations. Although crystal structures of HIV-1 show one Mg^2+^ ion in the RNase H active site [5], more recent structures of the more closely related *B. halodurans* and human RNases H show two magnesium ions [11,12], which is also supported by biochemical evidence of related enzymes [16]. This has led to the general acceptance of a two-metal ion mechanism for retroviral RNase H hydrolysis [11,12,17]. In brief, a two-metal ion mechanism requires that metal ion A activates a water molecule as a nucleophile and moves towards ion B, bringing the nucleophile in close proximity to the scissile bond, while metal ion B destabilizes the substrate-enzyme interaction and lowers the energy barrier to product formation (Figure 3) [18]. Ions A and B are involved in the stabilization of the transition state and product release. In order for hydrolysis to occur, the metals ions are likely coordinated at a distance of 3.5 to 4 Å from each other, possibly with some degree of flexibility (Figure 3) [18]. This scenario is exploited by small molecules used to inhibit RNase H activity (discussed below).

The homologous HIV-2 RT shows markedly reduced RNase H activity (10-fold). This discrepancy in activity has been shown to be due to a single residue (Q294) in the catalytically inactive p54 subunit, which is the structural equivalent of the p51 subunit in HIV-1 RT [19,20]. Mutagenesis of Q294 to P294 as in the WT HIV-1 RT shows an increase in RNase H activity comparable with that of HIV-1 RT [19]. Interestingly, all other mutations that have been tested at that position have also increased the RNase H activity of HIV-2 RT [19]. Q294 is highly conserved in HIV-2 isolates, as well as the related SIV_smm_ model [19]. P294 is seen in approximately 80% of sequenced isolates of HIV-1, while Q294 appears only about 1% of the time in HIV-1 and the recombinant mutant shows reduced RNase H activity [19]. Although a structural or mechanistic explanation so far remains elusive, it is interesting to note that the p51 thumb is implicated in inhibitor binding in HIV-1 (see below).

## 3. Substrate Binding

The nucleic acid binding cleft encompasses the distance between the active sites (∼60 Å) and can accommodate 17 base pairs of DNA/DNA, and 18 base pairs of RNA/DNA [21]. When RT is bound to a DNA/DNA substrate, the nucleic acid adopts A-form geometry near the polymerase active site, and B-form geometry near the RNase H active site [6]. However, in the context of an RNA/DNA substrate the nucleic acid adopts an H-form geometry that is neither A- nor B-form [7]. Multiple contacts are made between enzyme residues and the 2′-OH groups in RNA/DNA substrates that are not available on DNA/DNA substrates [5–7]. This likely contributes to tighter enzyme binding to RNA/DNA than DNA/DNA [22]. In order for the RNA template to be cleaved in the same complex that synthesizes DNA, the two activities of RT must be precisely coordinated. This is in part accomplished by a structural motif, referred to as the RNase H primer grip [7]. This network of amino acids makes contact with the DNA primer, and helps to determine the trajectory of the substrate in the context of the RNase H active site [7]. Residues of the RNase H primer grip involve G359, A360 and H361 of the connection domain, T473, Q475, K476, Y501 and I505 of the RNase H domain, as well as K395 and E396 of the p51 thumb subdomain [7]. The RNase H primer grip is mostly conserved among other RNases H including *E. coli* and MLV [7,9,23]. Likewise, mutations in the RNase H primer grip are deleterious to RNase H activity, presumably because the mutant enzymes are unable to position the substrate on a trajectory that is conducive to RNase H cleavage [24,25]. Primer grip mutants also lower the rate of DNA synthesis and lower virus infectivity [25–27].

**Figure 4.**
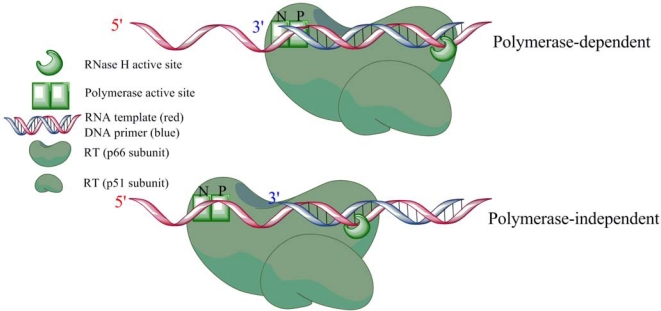
HIV RT can exist in two distinct binding modes when bound to a nucleic acid substrate. The polymerase-dependent mode is characterized by the polymerase active site being in contact with the 3′ primer terminus. All other possible conformations are considered polymerase-independent.

HIV-1 RNase H has two major distinct modes of activity, namely polymerase-dependent and polymerase-independent (Figure 4). RT will position itself with the polymerase active site at the 3′ end of the DNA primer, ready to accept an incoming nucleotide if given the opportunity. Cleavage in this situation will result in a cut on the RNA template, 18 base pairs (bp) upstream from the polymerase active site and the properly positioned 3′end of the primer. Per definition, this specific cut is known as polymerase-dependent RNase H activity [21,28–32]. In the presence of deoxyribonucleotides (dNTPs) RT will advance and RNase H will eventually continue to cut the template; however, the rate of RNase H cleavage is approximately seven times slower than polymerization [33]. In this sense, the polymerase and RNase H activities are spatially, but not temporally coordinated during polymerase-dependent activity.

**Figure 5.**
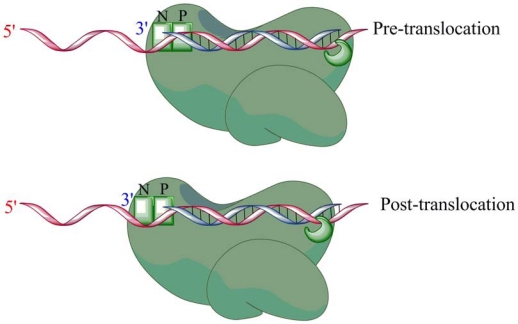
Polymerase-dependent binding can occur in two distinct positions. Post-translocation, where the 3′ primer terminus occupies the P site, leaving the N site available for nucleotide binding, or pre-translocation, where the N-site is occupied by the 3′ primer terminus and the incoming nucleotide is blocked by the primer terminus. An RT enzyme bound in a polymerase-dependent mode is in thermodynamic equilibrium between both pre- and post-translocational positions. The equilibrium is sequence-dependent.

There are two structurally distinct modes for RT to bind a primer/template substrate that depend on the translocational status (Figure 5) [34,35]. Pre-translocation is defined as the position where the 3′-end of the DNA primer is still occupying the nucleotide binding site (N-site), immediately after nucleotide incorporation. RT can move downstream by one nucleotide, so the nucleotide binding site is free to accept an incoming nucleotide, while the 3′ primer terminus now occupies the priming (P) site. This position is defined as post-translocation. Biochemical evidence suggests that RT can oscillate back and forth between pre- and post-translocational positions in a thermodynamic equilibrium that is sequence specific [34–38]. Polymerase-dependent RNase H cleavages therefore will exist as a population of two cleavages, each one representing either the pre- or post-translocational cleavage [39]. Binding of the next templated nucleotide will trap the enzyme in the post-translocational conformation, and binding of the pyrophosphate (PPi) analog PFA (phosphonoformic acid) traps the complex pre-translocation [38, 40, 41]. By trapping the enzyme in a pre- or post-translocational position, it has recently been shown that RT can simultaneously engage the nucleic acid substrate at both the polymerase and RNase H active sites [39].

RNase H activity where the 3′-end of the DNA primer is not occupying the polymerase active site is referred to as polymerase-independent RNase H activity. Polymerase-independent cleavages can occur internally, or they depend on the precise positioning of the 5′-end of RNA fragments relative to the polymerase active site [42]. The former refers to cleavages on long stretches of RNA/DNA that are too far from any terminus to be directed by nucleic acid ends, while the latter refers to cleavages that are directed by the 5′-end of the RNA template. It appears that these internal cleavages are affected by the nucleic acid sequence surrounding the cleavage site, as certain nucleotide positions in the vicinity of the scissile phosphate show a preference for specific nucleotides, although exactly what structural effect these sequence preferences may have and how it specifically directs RNase H cleavages is as yet unknown [43]. The other form of polymerase-independent RNase H cleavage is called 5′-end directed cleavage, and occurs on fragments of RNA recessed on a longer DNA strand [44]. The polymerase domain associates with the substrate near the 5′ end of the RNA. Cleavages of this type occur in a “cleavage window” of approximately 13–19 nucleotides from the 5′ termini of the RNA fragment [45].

Recent evidence suggests that the sequence specificity for internal RNase H cleavages are mostly shared by polymerase-dependent and –independent cleavages, including 3′ DNA- and 5′ RNA-end directed cleavages. Essentially all RNase H cuts show a preference for certain nucleotides at positions −4, −2 and +1 (where the scissile phosphate is between positions −1 and +1, and negative and positive positions proceed 5′ and 3′ from the scissile phosphate, respectively) [46]. Whether this preference is specifically for the RNA base or DNA base is unknown, as is the mechanism by which this preference occurs.

RT makes the decision of whether to bind as a polymerase or an RNase H based on the structure and composition of the nucleic acid substrate itself [31,47], and on the presence and absence of ligands. As previously stated, a recessed DNA 3′ primer end will be bound by the polymerase whether or not the template is DNA or RNA, whereas an RNA fragment recessed on a DNA template will be degraded with the polymerase domain associating with the substrate near the 5′ RNA end. Chimeric primers *i.e.*, RNA-DNA/DNA hybrids are recognized differently by RT depending on the relative length of both the RNA and DNA portion of the primer [31]. The part of the primer that is in contact with the C-terminal portion of RT near the RNase H domain is instrumental in determining the binding orientation of the enzyme, and thus the preferred activity *i.e.*, polymerization or RNA hydrolysis [47]. The single most important nucleotide in determining the activity of RT on a chimeric RNA-DNA primer is the primer nucleotide in contact with the RNase H primer grip, in particular residues T473 and Q475 [47].

## 4. Role of RNase H in (+)-strand priming

**Figure 6.**
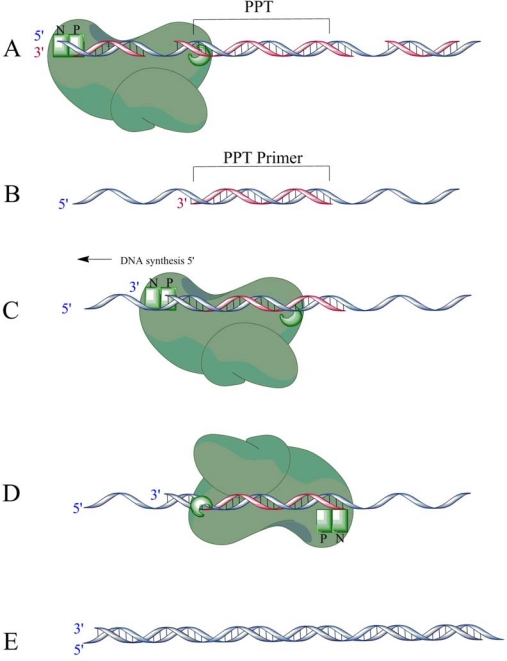
**A** During (−)-strand DNA synthesis, the PPT region of the RNA genome is resistant to RNase H cleavage, while a portion of the RNA genome is degraded concomitantly with (−)-strand DNA synthesis. Here, a specific cleavage is made to create the PPT primer. **B** The RNase H-resistant PPT sequence forms the primer for (+)-strand DNA synthesis when the rest of the genome is completely degraded by RNase H. **C** The RNA primer is extended 12 nucleotides, **D** then RT pauses and changes orientations to a polymerase-independent binding mode in order to cleave at the DNA:RNA junction, and remove the PPT primer. **E** The 12-mer fragment is extended toward the 5′ end of the (−)-strand DNA (see Figure 1F). After the second strand transfer event, the (+)-strand DNA is fully synthesized resulting in a fully double-stranded provirus. Adapted from [49].

A purine-rich sequence (5′ AAAAGAAAAGGGGGG 3′) called the polypurine tract (PPT) located just 5′ to the U3 sequence of the genome is resistant to RNase H cleavage and remains intact after the rest of the viral RNA has been removed (RNase H makes a specific cut following the 6th “G” residue to define the 3′ end of the PPT primer)(Figure 6A and B) [48–52]. The 3′-end of the newly formed RNA primer is extended by RT to position +12 downstream from the PPT RNA (Figure 6C). RT then pauses, re-orients itself and makes a specific cleavage at the PPT-U3 junction (Figure 6D) [49]. This cleavage is important as it defines the end of the U3 LTR, which is used as a substrate by the viral integrase. PPT primers with aberrant cleavages or that improperly begin DNA synthesis are not used efficiently and reverse transcription does not proceed [53]. In order for RT to successfully initiate (+)-strand DNA synthesis, RT must bind as a polymerase to extend the primer and as an RNase H to cleave at the RNA-DNA junction. As the active sites of RT are simultaneously positioned on opposite strands of the nucleic acid, RT must recognize its position on the genome and dynamically change its binding orientation appropriately for the task at hand. Thus, the primer removal reaction requires polymerase-independent RNase H activity.

In general, RT does not efficiently extend RNA primers with two exceptions: the (−)-strand or tRNA^lys3^ primer, and the (+)-strand or PPT primer. The PPT is structurally distinct in several ways that contribute to its resistance to RNase H cleavage. The PPT has an unusually narrow minor groove, and due to extensive contacts between the DNA primer and the RNase H primer grip, only substrates with a certain minor groove width appear to have appropriate access to the RNase H active site [7].

A co-crystal of RT together with an RNA/DNA PPT substrate shows that the trajectory of the substrate is “missing” the catalytic residues of the active site by about 3 Å, the same as the apparent narrowing of the minor groove [7]. The PPT has other unusual structural features such as an “unzipping” of the primer/template just downstream of the RNase H active site. An unpaired base on the template is followed by a frameshifted A:T pair and a mismatched G:T pair, followed by another unpaired base on the primer strand to restore the register [7]. The next base pair is adjacent to the scissile phosphate, and together with the next 3 base pairs upstream are in frame, although the bases are more widely separated [7]. This could also affect RNase H cleavage. It has also been hypothesized that during polymerization, the stiffness of the A-tracts in the PPT combined with the flexibility of the G:C base pair separating them could cause pausing of RT as it attempts to induce a 40° bend characteristic of nucleic acids bound to a wide variety of polymerases (including RT), which could allow time for RT to re-orient itself into an RNase H-competent mode and cleave at the PPT-U3 junction [7]. However, more research is required to confirm this hypothesis. Recent evidence has suggested that the initiation of (+)-strand synthesis is preferentially inhibited by non-nucleoside analog RT inhibitors (NNRTIs) that bind in close proximity to the nucleotide binding site, highlighting the importance of (+)-strand initiation in the development of potent antivirals [54].

## 5. Role of RNase H in strand transfer and (-)-strand primer removal

Reverse transcription in retroviruses is initiated by cellular tRNAs that are packaged into the virion during assembly, sometimes along with the corresponding tRNA synthetase. For HIV-1, human tRNA^lys3^ base pairs with its 18 3′-terminal residues with the primer binding site (PBS) near the 5′ end of the viral genome forming an RNA/RNA duplex. This primer is extended to the 5′ end of the genome producing the minus strand strong-stop DNA ((−)ssDNA). The (−)ssDNA must now dissociate from the genomic template and re-associate either with repeat (R) sequences at the 3′ end of either the same genome (Figure 1C) (intramolecular strand transfer) or the other copy of the genome present in the virion (intermolecular transfer), with both types of transfers happening at approximately equal frequency [55,56]. In order for the aforementioned dissociation of the (-)ssDNA occur, the RNA template must be degraded by RNase H cleavage. Models of the strand transfer reactions have been reviewed elsewhere [55].

The rates of strand transfer and RNase H activity are well correlated. Experiments with RNase H-deficient RT enzymes show that (-)ssDNA is formed, but strand transfer never occurs. Similarly if RNase H activity is moderately inhibited, strand transfer is also inhibited. The genomic template must be degraded to provide an available DNA sequence for an invading RNA template. If polymerization is arrested, with chain terminating nucleotides for example, strand transfer rates increase [57]. This is likely due to the relatively slower RNase H activity having enough time to make more cleavages due to the pausing of the enzyme, and this in turn allows dissociation of cleaved genomic fragments and invasion by a new template [58]. This type of pausing-induced RNase H activity is polymerase-dependent. While other RT enzymes not involved in polymerization can bind and cleave RNA fragments in a 5′-end directed or internal cleavage mode, they are unlikely to be affected by pausing of the polymerizing RT. The question is then raised of whether strand transfer requires polymerase-dependent cleavage or polymerase-independent cleavage, or both? *In vivo* experiments with MLV have demonstrated that a mutant RT enzyme with RNase H activity eliminated and also diminished polymerase activity does not allow for strand transfer, as expected. However when that system is supplemented with a polymerase-negative mutant with a fully functional RNase H (pol(−)/rnh(+)), strand transfer is only marginally increased [59]. It is assumed by the authors that the pol(−)/rnh(+) mutant is participating in mostly polymerase-independent RNase H activity, as the polymerizing RT will be occupying the 3′-end of the DNA primer, without being able to contribute any polymerase-dependent RNase H activity. The interpretation of these data by the authors suggests that in the absence of polymerase-dependent RNase H activity, polymerase-independent activity is inefficient. Also, that in the context of (−)ssDNA strand transfer, both polymerase-dependent and – independent activities are required for completion of reverse transcription [59]. However, as noted by the authors, it is possible that a pol(−)/rnh(+) RT is binding occasionally at the 3′-end of the primer and providing polymerase-dependent cuts. Telesnitsky and Goff showed that MLV can successfully replicate when the polymerase and RNase H activities are provided on different enzymes, but again this system cannot completely discriminate between polymerase-dependent and –independent cuts, since polymerase-negative mutants could bind to the 3′-end of the primer and induce polymerase-dependent cuts [60]. It is therefore unclear whether both modes of RNase H activity are required for successful strand transfer, however they do appear to have slightly differing roles.

In HIV-1 the tRNA primer is incompletely removed, with one rA:dT base pair remaining attached to the U5 terminus of the double-stranded DNA [56,61]. This rA is one of two bases removed during the 3′-end processing activity of the viral integrase. In most retroviruses, the sequence of the U5 or PBS regions do not affect cleavage specificity, however in HIV-1, the U5 sequence (G immediately 3′ of the conserved CA dinucleotide) is pivotal in determining RNase H cleavage specificity. Integrase has been shown to recognize diverse structures as substrates for 3′ processing and strand transfer [62]. It can remove both one and three nucleotides adjacent to the conserved CA at the end of U5, however it is not efficient. This is perhaps not surprising since the related RNase H from HIV-2 normally removes three nucleotides before the CA dinucleotide [63]. Studies suggest that mutation of the CA dinucleotide results in DNA ends that are inefficiently used by integrase and reduce viral titer *in vivo* [63]. However, due to the flexibility of integrase in its ability to use substrates of various lengths for 3′-end processing, it remains unclear if the disruption of cleavage specificity by RNase H by small molecules or other techniques is practically useful as a therapeutic option.

## 6. Role of RNase H activity in drug resistance

All established drugs that target HIV-1 RT have binding sites either at the polymerase active site, or the non-nucleoside binding pocket, a hydrophobic depression created by the binding of non-nucleoside reverse transcriptase inhibitors (NNRTIs) and located near the base of the p66 thumb subdomain, about 10 Å from the polymerase active site [4,64,65]. It is therefore not surprising that most of the major known resistance-conferring mutations are also located in the vicinity of the polymerase active site. However, recent studies have shown that mutations in the connection and RNase H domains can affect the susceptibility of RT to NNRTIs and nucleos(t)ide reverse transcriptase inhibitors (NRTIs). Furthermore, RNase H activity itself is implicated in the mechanism of resistance to NRTIs such as 3′-azidodeoxythymidine (AZT), and also to NNRTIs such as nevirapine (NVP). For example, the NNRTI resistance mutation V106A showed a reduction in both 3′-end and 5′-end directed RNase H activity, which correlated with a decrease in viral fitness [66]. In contrast, the NNRTI-resistance mutant Y181C demonstrates an increase in RNase H activity, while still demonstrating decreases in viral fitness levels despite no change in polymerization processivity compared to wildtype [66]. AZT also selects for other connection/RNase H domain mutants such as A360I/V, A371V and Q509L [67–73].

The connection domain mutant N348I has been found in clinical isolates with treatment regimes that include both AZT and NVP [73], becoming the first resistance mutation selected outside of the polymerase domain that confers dual-class resistance. It was proposed that when RNase H activity degraded the RNA template of an AZT-terminated primer, the chain-termination became permanent, as the template fragments would dissociate and the single-stranded AZT-terminated primer is not a substrate for the excision machinery. Therefore, decreased RNase H activity could act to increase the half-life of the template, allowing more time for RT to excise AZT at the primer terminus [67,74–78]. This hypothesis was supported by a study that linked decreased RNase H activity to increased rates of excision [73], and Ehteshami *et al.* provided a plausible biochemical mechanism [79]. As N348I and A360V often appear along with other thymidine analog mutations (TAMs) in the clinic, they both show resistance to AZT. RT enzymes containing N348I and A360V reduce RNase H activity by selectively dissociating from RNase H-competent (polymerase-independent) complexes [79]. N348I had no effect on substrate binding of the polymerase domain, and A360V rescued a modest binding defect introduced by TAMs, which agrees with the observation that A360V appears later in therapy after the emergence of TAMs and N348I [79]. The A360V and N348I mutations also appear to increase processive DNA synthesis, which points to an additional RNase H-independent contribution to resistance [79].

The RNase H mutant Q509L likely operates in a similar mechanism, although it appears only rarely in therapy-experienced patient [67,80,81]. Also, G333D/E mutation is resistant to AZT and lamivudine (3TC) although it does not affect RNase H activity. This mutation as well as others such as E312Q, G335D, V365I, A371V and A376S that affect resistance to NRTIs and in some cases NNRTIs, have been reviewed [69,82].

## 7. Inhibitors of HIV-1 RT-associated RNase H activity

There are currently 25 compounds in clinical use to treat HIV [2]. 12 of those 25 compounds target RT. Despite having two distinct enzymatic activities that are both absolutely necessary for successful replication of the virus, all of the 12 RT-targeting compounds block the polymerase activity of RT. This extreme bias of drugs towards the polymerase activity could be attributed in part to the relatively flat surface topography of the RNase H domain, unlike the polymerase domain which contains mobile subdomains and hydrophobic pockets providing a foothold for small molecules. In response to this, research has focused on the RNase H active site itself, in particular the two metal ions coordinated by the DEDD motif described earlier. This has led to several prototype compounds that chelate the Mg^2+^ ions through a 3-oxygen pharmacophore originally designed for inhibition of the influenza endonuclease [83], as well as several other compounds acting through a different mechanism. RNase H inhibitors that utilize a cation-chelating mechanism as their mode of inhibition are referred to as active site inhibitors [16].

**Figure 7.**
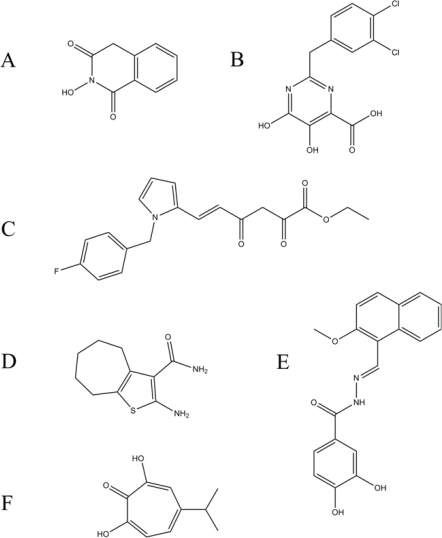
Examples of the main classes of small molecule inhibitors against HIV-1 RT-associated RNase H activity. **A** N-hydroxyimides. Developed from influenza inhibitors and among the first to use the 3-oxygen pharmacophore [84]. **B** Pyrimidinol carboxylic acid derivatives [85]. Potent inhibitors based on the successful scaffold of the metal-chelating active site inhibitors. **C** Diketo acid derivatives are also active in some cases against the viral integrase. RDS1643 shown here is the only diketo acid to have antiviral activity *in vivo* [86]. **D** The vinylogous ureas (NSC727447 pictured here) represent a different kind of inhibitor that binds allosterically near the p51 thumb [87]. **E** N-acyl hydrazones appear to bind to multiple binding sites depending on the specific inhibitor, including the RNase H domain and a site that overlaps the NNRTI binding pocket (DHBNH picture above) [88]. **F** Hydroxylated tropolones (β-thujaplicinol pictured here) are the subject of several studies that have provided the basis for a biochemical mechanism of inhibition by active site RNase H inhibitors [39,89,90].

The first of such compounds designed for the inhibition of HIV-1 RNase H activity were N-hydroxyimides (Figure 7A) [16]. These compounds were active against the isolated HIV RNase H domain and were highly selective as compared with *E. coli* RNase H [16,84]. The metal ions were separated by a distance of 4–5 Å and chelated by three oxygens, with the N-hydroxyl acting as a bridging oxygen contacting both metal ions [16,84]. A crystal structure of the isolated RNase H domain with a bound N-hydroxyimide inhibitor in the presence of Mn^2+^ confirms the binding orientation and provides proof-of-concept for metal-chelating RNase H inhibitors [91].

Diketo acids also form a group of compounds initially developed against the structurally similar HIV-1 integrase (IN) [92,93]. As such, the first diketo acid inhibitor developed showed similar inhibition of full-length RT, the isolated RNase H domain, and IN with IC_50_ values for all three in the low micromolar range, although it had no effect on the polymerase activity of RT [94]. The diketo acid 4-[5-(benzoylamino)thien-2-yl]-2,4-dioxobutanoic acid (BTDBA), was shown to bind to the isolated HIV RNase H domain in a metal-dependent fashion by isothermal titration calorimetry [94]. It also did not appear to require the presence of the nucleic acid substrate for efficient binding [94]. The presence of the 3-oxygen pharmacophore suggests a similar mode of inhibition to the N-hydroxyimides. However, despite effectively inhibiting RNase H activity *in vitro*, this compound did not show inhibition of viral replication in cell culture [94]. Another diketo acid-based inhibitor, RDS1643, also specifically inhibited HIV RNase H activity while not affecting the HIV polymerase activity, or the RNase H activity of related enzymes such as *E. coli* and avian myeloblastosis virus (AMV) (Figure 7C) [86]. Unlike other active site inhibitors, RDS1643 inhibited viral replication in MT-4 cells at essentially the same IC_50_ as the *in vitro* assays (∼13 μM) [86]. Consistent with other diketo acids, binding of RDS1643 did not require the substrate for binding, but has a specific requirement for divalent cations. The mode of inhibition of RDS1643 has been determined to be reversible and non-competitive [86]. Experiments with diketo acid inhibitors were instrumental in showing proof-of-concept that RNase H inhibitors could be synergistic with NNRTIs in a full reverse transcription assay. Curiously, the two inhibitors are antagonistic in an RNase H assay, and additive in an RNA-dependent DNA polymerase assay [95].

A third structural scaffold for the 3-oxygen pharmacophore is represented by the hydroxylated tropolones. One of the first such compounds described, β-thujaplicinol is a natural product derived from the plant *Thuja plicata* and is active at submicromolar concentrations against HIV-1 and HIV-2 RNase H, precluding the NNRTI-binding pocket as the binding site (Figure 7F) [89]. It is also a specific inhibitor of HIV RNase H, and is ∼30 and ∼250-fold less effective at inhibiting human and *E. coli* RNase H1 respectively, and showed no inhibition of the polymerase activity of RT [89]. However, β-thujaplicinol lacks antiviral activity in cell culture [89].

Recent studies by Beilhartz *et al.* have elucidated the underlying biochemical mechanism of β-thujaplicinol. β-thujaplicinol appears unable to bind to enzyme-substrate (E-S) ternary complexes stabilized by either an incorporated dideoxynucleotide (ddNTP) and the next templated nucleotide (post-translocation) or the pyrophosphate analog PFA (pre-translocation) [39]. Furthermore, in order-of-addition experiments with very high enzyme to substrate ratios, no inhibition of RNase H activity was observed unless the enzyme and β-thujaplicinol were allowed to pre-incubate in the presence of Mg^2+^, and the reaction was started with the primer/template [39]. These data strongly suggest competition between the primer/template and the inhibitor, while providing support for a metal ion-dependent binding mode. The order-of-addition data also show that the substrate is eventually cleaved to the same level as without inhibitor, suggesting that the substrate binds tighter and will eventually displace β-thujaplicinol in the RNase H active site [39]. This competitive mode of inhibition is in apparent contrast to previously published results on β-thujaplicinol and other active site inhibitors [86,89]. In order to reconcile this, we proposed that β-thujaplicinol is ineffective at inhibiting the primary RNase H cut, but instead binds to the product complex and inhibits further cleavages [39]. In this context, the primary cut refers to the first cut made on the substrate. On a substrate that allows polymerase-dependent cleavage, this would be at positions representing pre- and post-translocational stages. In contrast, on a substrate that allows polymerase-independent cleavage, the position of the primary cut would vary. Results from a polymerase-independent substrate mimicking the (-)-strand primer removal reaction support this hypothesis and show strong inhibition of secondary cuts, while efficiency of the primary cut is not significantly affected [39].

**Figure 8.**
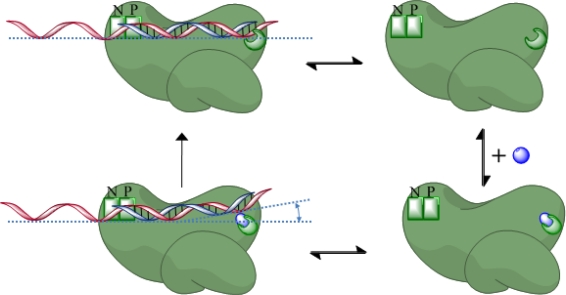
A schematic of possible models of active site inhibitor binding, based on studies with the tropolone derivative β-thujaplicinol (blue sphere) [39,90]. Evidence suggests that the inhibitor is unable to bind to an enzyme-substrate (E-S) complex (top left), only to free enzyme forming (top right) and enzyme-inhibitor (E-I) complex (bottom right). However, the substrate might be able to bind to this E-I complex, forming an E-S-I complex that is not productive with respect to RNase H cleavage (bottom left). As suggested by Himmel *et al.,* the inhibitor occupies the position normally claimed by the scissile phosphate [90]. As such, it is possible that the substrate undergoes a change in trajectory in relation to the scissile phosphate and the RNase H active site [39]. Then eventually, the inhibitor dissociates and RT is allowed to cleave the uninhibited substrate (E-S complex, top left).

Recently, Himmel *et al*. presented the crystal structure of RT complexed with β-thujaplicinol at a resolution of 2.80Å [90]. The structure presented confirms the metal-ion coordination of β-thujaplicinol while also confirming that the target is indeed the RNase H active site [90]. Superposition of the RT- β-thujaplicinol structure with the structure of a human RNase H1 complexed with an RNA/DNA substrate suggests that the bound β-thujaplicinol would invoke a steric clash with both the scissile phosphate and the water molecule that would instigate a nucleophilic attack during RNA hydrolysis (see above) [90]. This is in good agreement with Beilhartz *et al*., who suggest that competition between the bound inhibitor and the substrate is the mechanism of inhibition [39]. Himmel *et al.* present biochemical evidence that the primer/template could bind to a pre-formed RT- β-thujaplicinol complex even if the reverse is not possible, as shown by Beilhartz *et al.* This model would likewise be in agreement with classical non-competitive kinetics measured under steady-state conditions [39,89,90]. In this case, dissociation of the active site inhibitor may be hindered by the bound nucleic acid substrate in the context of an enzyme-inhibitor-substrate (E-I-S) complex. The nature of changes in RT-primer/template interaction has yet to be elucidated in this context. The putative steric clash between the nucleic acid substrate and the bound inhibitor suggests a change in the trajectory of the substrate, in relation to the RNase H active site (Figure 8).

The combined data strongly suggest that the screening of potential RNase H inhibitors under classic steady-state conditions requires stringent secondary screening assays. Measurements under pre-steady-state conditions, or experiments with stable ternary complexes would help to identify compounds that may block primary cuts. The question of whether a putative RNase H inhibitor must inhibit primary cuts, secondary cuts, or both in order to be effective remains to be answered. The observation that β-thujaplicinol does not block primary cleavages also offers a possible explanation as to why many active site inhibitors do not show antiviral activity *in vivo*, as primary cuts could be sufficient to allow successful reverse transcription. The combination of a high-resolution structure and detailed biochemical evidence is an extremely important advancement in the development of potent, safe inhibitors against RNase H, and β-thujaplicinol appears to be a critical step toward this end.

A recent study has introduced a fourth scaffold of the 3-oxygen pharmacophore, pyrimidinol carboxylic acid derivatives (Figure 7B) [85]. Compounds of this class show IC_50_ values in the low to submicromolar range against HIV RNase H activity, and were selective for the viral enzyme compared to human RNase H1 [85]. Order-of-addition experiments support a metal-dependent binding mechanism for these compounds, in agreement with experiments using β-thujaplicinol [39,85]. One of the pyrimidinol carboxylic acids was successful crystallized with Mn^2+^ and the isolated HIV RNase H domain [85]. The carboxyl group coordinates metal ion B, while metal ion A (responsible for activating a water molecule for nucleophilic attack) is coordinated by the two phenolic oxygen atoms of the bound inhibitor [85]. Furthermore, possible π-interactions between the inhibitor and H539 are observed in the crystal structure, although what effects mutagenesis at position 539 may have on inhibitor binding is unknown [85].

Despite the effort involved in the development of active site inhibitors, they are not the only compounds that have been described as RNase H inhibitors. Several other classes of compounds have been shown to inhibit HIV-1 RNase H and are characterized as allosteric inhibitors, as they do not bind at the RNase H active site. Most notably among these are the hydrazones and vinylogous ureas.

N-acyl hydrazones were originally described as dual inhibitors of HIV RNase H and polymerase activities. The first of such compounds (N-(4-tert-butylbenzoyl)-2-hydroxy-1-naphthaldehyde hydrazone) or BBNH was observed to have metal ion-chelating abilities, while lacking the 3-oxygen pharmacophore of the active site inhibitors [96]. Also, BBNH inhibited *E. coli* RNase H1 which, in contrast to the 2-metal ion mechanism of HIV RNase H, uses a 1-metal ion activation/attenuation mechanism, further distancing itself from the active site inhibitors [16,97,98]. BBNH also failed to inhibit the RNase H activity of HIV-2 RT, which does not possess the NNRTI-binding pocket of HIV-1 RT [96]. However, BBNH was effective at inhibiting NNRTI-resistance mutants in HIV-1 RT, suggesting two distinct binding sites for BBNH: one in the RNase H domain and one that overlaps with the NNRTI-binding pocket [96]. It was also shown that BBNH impacts the stability of the p66/p51 heterodimer [99]. Modeling studies have suggested that Y501 is important for BBNH binding [100]. Mutants Y501W and Y501R were both resistant to BBNH-mediated inhibition of the RNase H activity of RT, but remained sensitive to inhibition of the polymerase activity, which strongly supports the two-binding site hypothesis [100]. Other variations of BBNH inhibit only the polymerase activity of RT (4-t-butylbenzoyl)-2-hydroxy-1-salicylyl hydrazone (BBSH) while another derivative inhibited only the RNase H activity (4,N,N-dimethylaminobenzoyl)-2-hydroxy-1-naphthyl hydrazone (DABNH). Of these, BBSH also affected dimer stability like its predecessor BBNH [99]. A crystal structure of yet another hydrazone derivative, ((E)-3,4-dihydroxy-N’-((2-methoxynaphthalen-1-yl)methylene)benzohydrazide) (DHBNH), shows the inhibitor bound near the NNRTI-binding pocket, in contact with residues W229 and D186 (Figure 7E) [88]. This is in agreement with one of the proposed binding sites for BBNH. However, DHBNH also weakly inhibits the isolated HIV RNase H activity although two binding sites are not observed in the crystal structure [88]. It is possible that DHBNH shares the same two binding sites as BBNH. It is not known whether DHBNH impairs dimer stability. More research is required to elicit the exact binding sites of this class of RT inhibitor.

Another class of RNase H inhibitors that bind in an allosteric manner is the vinylogous ureas. These compounds are thought to bind near the p51 thumb subdomain, based on protein footprinting/mass spectroscopy experiments [87]. The p51 thumb is in contact with the p66 RNase H domain, and mutations in this region have been known to affect RNase H activity [101,102]. Mutations in the RNase H primer grip residues Y501 and T473 show differing effects [87]. The mutant Y501Bp-Phe which represents the mutation of residue 501 from tyrosine to benzophenyl-phenyalanine (an unnatural amino acid), confers resistance to the compound NSC727447 (IC_50_ values 6.6 μM for Y501 to 196.7 μM for Bp-Phe501) (Figure 7D) [87]. However, the mutation T473C increases sensitivity of the enzyme for NSC727447 by 50-fold, and showed a significantly reduced affinity of T473C for its substrate in the presence of NSC727447 [87]. The effects of the inhibitor on primer grip mutants support the hypothesis that NSC727447 binds near the p51 thumb.

The development of potent allosteric inhibitors provides new options for potential inhibitor binding sites, such as the p51 thumb subdomain. This combined with the development of potent active site inhibitors could result in a powerful new combination of therapeutic options in the treatment of HIV infection. The field of HIV RNase H drug development has made significant advances. The emergence of crystal structures with bound inhibitors are an encouraging development and more are urgently needed, especially including the bound nucleic acid substrate. The field is also patiently awaiting the first RNase H inhibitor with (potent) antiviral effects. The selection of resistance in cell culture is viewed as an important milestone in this regard.

## 8. Conclusion: Discovery and development of *bona fide* RNase H inhibitors

There have been many other compounds described in the literature that show apparent inhibition of RNase H activity, including natural products such as various quinones and napthoquinone derivatives, although many of these suffer from a lack of potency [103,104]. DNA and RNA aptamers have recently been shown to be potent and selective inhibitors of HIV-1 RT, and its RNase H function, in cell-free assays [105–107]. Their utility in cell-based assays is currently under investigation. RNA-based RNase H inhibitors can potentially compete with the nucleic acid substrate for the same contacts; however, the mechanism of action of small molecule inhibitors remains often elusive and their effects on RNase H cleavage can be indirect. For instance, the pyrophosphate analog PFA that binds to the polymerase active site was also shown to reduce RNase H activity under steady-state conditions [95]. However, inhibition of RNase H cleavage is not seen under pre-steady-state conditions [39]. PFA traps and stabilizes the pre-translocated complex, which, diminishes the turnover of the reaction, which, in turn, translates into RNase H inhibition. Thus, the effect of PFA on RNase H cleavage is not direct and most likely irrelevant in biological settings. There is an excess of HIV-1 RT in the virion and frequent dissociation of the enzyme will also cause dissociation of PFA. Unliganded RT molecules may then bind to the nucleic acid substrate and RNase H cleavage would occur uncompromised. This is a fundamental difference to the aforementioned RNase H active site inhibitors that bind to free enzyme in the presence of divalent metal ions. It is therefore difficult to discern whether a given compound with apparent inhibitory effects on RNase H activity is a *bona fide* RNase H inhibitor unless it has been assessed in more detailed mechanistic studies. Pre-steady-state kinetics, order-of-addition experiments, and the use of polymerase active site mutants and/or the use of the isolated RNase H domain have been proven successful with respect to the characterization of RNase H active site inhibitors. The characterization of allosteric RNase H inhibitors requires in addition to these experimental approaches the determination of the binding site for the inhibitor.
